# Co-Option and Conflict: The Deep Evolutionary History of ZP-Domain Proteins from ECMs to Species Barriers

**DOI:** 10.3390/ijms27135866

**Published:** 2026-06-29

**Authors:** Natalia Bezborodkina, Daniil Smutin, Leonid Adonin

**Affiliations:** 1 Zoological Institute, Russian Academy of Sciences, 199034 St. Petersburg, Russia; 2 Faculty of Information Technology and Programming, ITMO University, 197101 St. Petersburg, Russia; 3 Institute of Biomedical Chemistry, 119121 Moscow, Russia; 4 Federal State Budget-Financed Educational Institution of Higher Education, The Bonch-Bruevich Saint-Petersburg State University of Telecommunications, 193232 St. Petersburg, Russia

**Keywords:** ZP (Zona Pellucida), ZP-domain proteins, egg coat evolution, molecular coevolution, phylogenetics, metazoan reproduction

## Abstract

The Zona Pellucida (ZP) and its structural analogs are evolutionarily ancient extracellular matrix components. These are essential for oocyte protection, species-specific gamete recognition, and prevention of polyspermy across Metazoa. Defined by the conserved ZP-domain—comprising ZP-N and ZP-C subdomains—these glycoproteins self-assemble into fibrillar matrices through tightly regulated polymerization. Mechanisms of the regulated polymerization involve furin cleavage, disulfide bonding, and hydrophobic interactions. Once considered a vertebrate innovation, the canonical ZP-domain—defined by its bipartite ZP-N/ZP-C architecture, eight conserved cysteine residues, and capacity for matrix polymerization—is now recognized as an ancient metazoan extracellular module, with homologs identified in basal lineages including Porifera, Cnidaria, and Placozoa. While ZP-like sequences have been reported in choanoflagellates such as *Salpingoeca rosetta*, these lack the complete canonical features and are considered distant structural relatives rather than true ZP-modules. There they function in cell adhesion and tissue integrity, suggesting an origin predating the evolution of specialized reproductive coats. Previous phylogenetic analyses across 97 metazoan species have revealed that vertebrate ZP genes arose from ancestral duplications of the canonical ZP-module. Accordingly, they give rise to eight subfamilies (*ZP1–ZP4*, *ZPD*, *ZPAX*, *ZPX*, *ZPY*), with lineage-specific expansions, losses, and pseudogenization reflecting adaptations to diverse reproductive strategies. Positive selection in sperm-binding regions of *ZP2* and *ZP3* drives a rapid adaptive evolution. It underscores coevolutionary arms races with sperm ligands, contributing to reproductive isolation and speciation. In invertebrates such as abalone and insects, ZP-domain proteins mediate analogous functions through lineage-specific elaborations, including tandem repeats and domain shuffling. Post-translational modifications, particularly glycosylation, fine-tune sperm receptor specificity and matrix stability. The functional transition from a general protective barrier in early metazoans to a sophisticated gamete recognition interface in vertebrates exemplifies modular evolution. This synthesis highlights the domain-level deep homology of ZP-domain proteins as a foundational element of metazoan extracellular matrices, repurposed through gene duplication, neofunctionalization, and selection to meet the demands of evolving reproductive modes. These insights bridge evolutionary biology, reproductive medicine, and developmental genetics. However, major gaps remain, including unresolved orthology between vertebrate and invertebrate ZP genes, the relative contribution of glycans versus protein backbone in sperm recognition, and the lack of functional evidence for canonical ZP-domain proteins in insects. Future studies integrating glycoproteomics, single-cell transcriptomics, and CRISPR-based models are needed to resolve these questions.

## 1. Introduction

The reproductive success of multicellular animals is fundamentally dependent on the integrity and functionality of the specialized extracellular matrix that surrounds the oocyte—the egg coat. This structure plays essential roles in gamete recognition, fertilization regulation, protection of the developing embryo, and prevention of polyspermy [1]. Among its components, the zona pellucida (ZP) and its structural analogs have attracted significant attention due to their evolutionary conservation and critical functions in species-specific sperm binding and fertilization [2].

ZP proteins are defined by the presence of a conserved ~260-amino-acid structural unit, hereafter referred to uniformly as the ZP-domain, which is composed of ZP-N and ZP-C subdomains and is central to the structural and functional organization of the egg coat across Metazoa [3,4]. This domain facilitates glycoprotein polymerization into a fibrillar matrix, serving as a selective barrier for sperm and a platform for gamete interaction [5,6]. Despite substantial sequence divergence, the ZP-domain is evolutionarily conserved across Metazoa, suggesting a shared ancestral origin dating back over 600 million years [7,8]. Comparative studies in basal chordates, such as cephalochordates, reveal polymorphic ZP proteins with distinct domain architectures, supporting an invertebrate origin for vertebrate ZP genes and highlighting early evolutionary innovations in gamete recognition systems [8].

According to previous phylogenetic analyses of ZP genes across 97 metazoan species, vertebrate ZP proteins—all of which contain the canonical bipartite ZP-N/ZP-C module—are classified into eight subfamilies, likely originating from a limited number of ancestral genes through repeated gene duplication and divergence [2,9]. Mammals typically retain four subfamilies (*ZP1–ZP4*), while lineages such as ray-finned fishes exhibit extraordinary diversification, with up to 33 ZP homologs in some species, reflecting differential gene retention and lineage-specific adaptations [2,10]. These evolutionary dynamics are further shaped by whole-genome duplications (WGD), particularly in teleosts, where duplicated ZP genes undergo subfunctionalization or neofunctionalization in response to selective pressures from sperm competition and reproductive isolation [11,12,13]. In certain teleost lineages, ZP gene expression has shifted from the ovary to the liver, enabling systemic delivery of egg-coat components and adaptation to external fertilization [10,14].

Previous maximum likelihood analyses, utilizing site-specific models such as M1a/M2a and M7/M8 implemented in *PAML*, have demonstrated elevated dN/dS ratios in mammalian ZP genes, indicating positive selection driven by sexual conflict, sperm competition, and the need for species-specific gamete compatibility [15,16,17]. These foundational studies, which rigorously controlled for potential methodological artifacts such as recombination, alignment errors, and relaxed selective constraints, have specifically identified rapid adaptive evolution in the sperm-binding interfaces of *ZP2* and *ZP3* across diverse mammalian lineages, including muroid rodents like Peromyscus and primates [15,16,17]. For example, the N-terminal region of *ZP2*, cleaved by ovastacin post-fertilization to block polyspermy, evolves rapidly, suggesting it is a hotspot for coevolutionary arms races with sperm ligands [18,19]. Similarly, in abalones, the ZP-N domain of the vitelline envelope receptor for lysin (VERL) contains tandem repeats that coevolve with the sperm protein lysin, exemplifying a molecular “arms race” that reinforces reproductive isolation [1,20,21]. Such coevolutionary dynamics underscore the role of ZP proteins in speciation and the maintenance of species boundaries [22,23].

Structural and functional divergence in ZP proteins is further modulated by post-translational modifications. Glycosylation patterns influence sperm receptor specificity and extracellular matrix stability, contributing to reproductive compatibility across taxa [24,25]. Disulfide bonding and transglutaminase-mediated cross-linking, particularly in teleosts, provide alternative mechanisms for egg-coat hardening post-fertilization, contrasting with the proteolytic remodeling observed in mammals [6,26]. These modifications fine-tune the physical and biological properties of ZP proteins, ensuring both structural resilience and dynamic regulation of fertilization [3,27].

Despite advances in structural biology and genomics, the precise mechanisms of ZP protein polymerization, domain organization, and evolutionary trajectory remain incompletely understood. While the ZP-domain was once considered a single structural unit, it is now recognized as comprising two independently folding modules (ZP-N and ZP-C), complicating phylogenetic interpretations [5]. Furthermore, phylogenetic evidence suggests that vertebrate ZP genes are not orthologous to invertebrate ZP-like proteins, implying multiple independent origins or deep divergence [2]. This lack of clear orthology, combined with extensive lineage-specific gene loss and pseudogenization (e.g., *ZPAX* and *ZPD* in mammals), challenges efforts to reconstruct the evolutionary history of the ZP superfamily [9].

The functional significance of ZP proteins extends beyond basic reproduction. Mutations in human *ZP1*, *ZP2*, or *ZP3* are associated with infertility due to defective matrix assembly [28,29], while species-specific glycosylation patterns contribute to reproductive isolation [30]. These insights have practical implications; immunization with ZP glycoproteins has been explored as a contraceptive strategy in wildlife management and conservation [29]. Moreover, emerging technologies—including cryo-electron microscopy, next-generation sequencing, and integrative proteomics—are enabling high-resolution analyses of ZP architecture and evolution, revealing how subtle molecular changes can influence fertility and evolutionary trajectories [6,7,31,32].

This review aims to synthesize evidence on the evolutionary history, structural complexity, and functional diversification of canonical ZP proteins across Metazoa, while distinguishing them from ZP-like domains found outside this clade. Previous studies have suggested and available evidence indicates that ZP proteins evolved from a common ancestral extracellular matrix component, with the core ZP-domain preserved over deep evolutionary time and subsequently modified through gene duplication, domain shuffling, and positive selection to meet taxon-specific reproductive demands [1,2,14]. By integrating genomic, proteomic, and phylogenetic data from basal invertebrates to vertebrates reported in the literature, we summarize the reconstructed phylogeny of ZP proteins, characterize domain architectures and post-translational modifications described in previous research, highlight the evolutionary forces driving diversification, and assess the role of structural divergence in gamete recognition and reproductive isolation based on available evidence [8,14]. This synthesis will contribute to a deeper understanding of reproductive molecular evolution and inform applications in fertility medicine, contraception, and evolutionary developmental biology [20,33].

## 2. ZP-like Proteins and Structures in Metazoans

To accurately trace the evolutionary trajectory of these proteins, it is crucial to establish a clear conceptual framework distinguishing between domain-level homology and gene-family orthology. Importantly, within this framework we further distinguish the canonical ZP-module—a metazoan synapomorphy defined by its bipartite ZP-N/ZP-C architecture, eight invariant cysteine residues, and demonstrated polymerization capacity—from ZP-like domains identified in unicellular holozoans such as the choanoflagellate *Salpingoeca rosetta*. The latter lack complete canonical features and experimental evidence for matrix polymerization, and are therefore treated as distant structural relatives rather than true ZP-modules. The ZP-domain represents an ancient, conserved domain-level homology, a bipartite ZP-N/ZP-C structural module whose immunoglobulin-like β-sandwich architecture and disulfide-bonded core are recognizable across distantly related extracellular proteins, including egg-coat components and non-reproductive ECM molecules [5,17,23]. Homology at this level indicates descent from a common ancestral ZP-N/ZP-C module, not necessarily from a common ancestral gene encoding a particular ZP family member. By contrast, gene-family orthology refers to 1:1 descent relationship among specific ZP genes or subfamilies (e.g., *ZP1*, *ZP2*, *ZP3*, *ZP4*, *ZPAX*, *ZPD*), which have been extensively reshaped by lineage-specific duplications, losses, and pseudogenization in vertebrates and other metazoans [2,8]. As a result, vertebrate ZP genes can share domain-level homology with invertebrate ZP-like genes while lacking simple orthologous correspondences [2,21].

This ancient ECM module has been repeatedly co-opted into reproductive contexts, such that ZP-domain proteins form the structural and functional core of egg coats in vertebrates, cephalochordates, mollusks, and other lineages [3,8,14,21]. Co-option implies the recruitment of a pre-existing ZP-N/ZP-C scaffold into gamete recognition and egg-coat polymerization, often accompanied by tandem domain expansion and neofunctionalization of ZP-N repeats [3,14,17,23]. However, similar roles of ZP-domain proteins in insect vitelline envelopes, molluscan VERL-containing envelopes, and vertebrate zonae pellucidae should be treated as functional analogy or convergence unless robust phylogenetic and synteny analyses demonstrate shared gene-family ancestry [2,3,8,21]. Functional similarity in sperm binding or egg-coat architecture alone cannot be taken as evidence of direct gene orthology.

The evolution of extracellular matrix (ECM) components has been central to the emergence of multicellularity and complex tissue organization in animals. Among these, the ZP-domain—a module originally identified in vertebrate egg-coat glycoproteins—has been found across diverse metazoan lineages [3]. Although the mammalian zona pellucida is the best-characterized example, the structural framework provided by the ZP-domain represents an ancient innovation that has been co-opted for multiple roles in early-branching taxa. This section examines the presence and functional significance of ZP-domain-containing proteins in basal metazoans, focusing on Porifera, Cnidaria (Hydra, *Aurelia aurita*, *Nematostella vectensis*), and selected invertebrates such as Platyhelminthes and Rotifera. This section highlights how these proteins contribute to cell adhesion, tissue organization, and reproductive biology, underscoring the domain-level deep homology that unites disparate metazoan lineages [14].

### 2.1. ZP-like Proteins in Porifera

Porifera, as the earliest diverging extant metazoans, possess a complex ECM despite their morphological simplicity. Genomic and transcriptomic analyses have identified putative ZP-domain-containing proteins in sponges such as *Amphimedon queenslandica*, even in the absence of a vertebrate-like zona pellucida [34]. The presence of conserved ZP-domain-encoding genes in the *A. queenslandica* genome suggests that these proteins may have been present in early metazoan ancestors [35]. In the choanoderm—the feeding layer of sponges—ZP-domain proteins are predicted to participate in ECM assembly, likely facilitating intercellular adhesion and contributing to tissue integrity [35]. However, it should be noted that these functions remain computationally inferred, and direct functional validation of ZP-domain proteins in poriferans is currently lacking.

Despite lacking a specialized egg envelope, sponges appear to retain canonical structural features of the ZP module, including conserved cysteine residues and predicted disulfide connectivity [2]. Bioinformatic predictions indicate that sponge ZP proteins possess the structural capacity to polymerize into fibrillar networks—a property that, if confirmed experimentally, would be critical for the assembly of higher-order ECM structures in more complex metazoans [2]. Their putative role may extend beyond adhesion to include cellular signaling and morphogenesis, potentially contributing to the transition from unicellularity to multicellularity [14]. The molecular conservation of the ZP-domain in *A. queenslandica* supports the hypothesis of domain-level deep homology, suggesting that core ECM components likely predate the divergence of complex animal lineages [3]. Thus, the presence of ZP-domain proteins in sponges points to a putative ancestral role in ECM structuring, which may have provided a molecular foundation for the subsequent evolution of reproductive matrices in later metazoans. However, all current evidence is based on genomic predictions and in silico sequence analysis; direct functional validation of ZP-domain proteins in Porifera (e.g., localization, polymerization assays, or genetic perturbation) remains limited, and their proposed functions remain hypothetical.

### 2.2. ZP-like Proteins in Cnidaria (Hydra, Aurelia aurita, Nematostella vectensis)

Cnidaria exhibit greater tissue specialization and reproductive complexity than sponges. ZP-like proteins are found in the envelopes surrounding eggs and larvae, indicating their co-option into reproductive roles. The ZP-domain is conserved in cnidarian species such as Hydra, *A. aurita*, and *N. vectensis* [14].

In the scyphozoan jellyfish *A. aurita*, a “contact plate” on the oocyte surface contains glycoproteins that cross-react with antibodies against mesoglein, a known ZP-domain protein [36]. This structure is hypothesized to mediate sperm recognition and binding, functionally reminiscent of vertebrate sperm-binding egg-coat components [37]. Proteomic studies confirm the presence of ZP-domain proteins in this structure and reveal essential post-translational modifications such as glycosylation [38].

In the sea anemone *N. vectensis*, multiple ZP-domain proteins have been identified in the jelly matrix surrounding oocytes. This matrix provides mechanical protection and may regulate species-specific sperm–egg interactions. Immunofluorescence reveals dynamic redistribution of these proteins during early development, suggesting roles in morphogenesis and metamorphosis [14].

In Hydra, ZP-domain-encoding transcripts are upregulated during oogenesis, and biochemical studies confirm their incorporation into the egg’s ECM [14]. Their polymerization contributes to the mechanical integrity of the envelope [39], and emerging evidence suggests broader roles in embryonic patterning and body plan formation. The remarkable structural conservation between cnidarian and vertebrate ZP proteins points to deep evolutionary conservation of ECM assembly mechanisms [3].

### 2.3. ZP-like Proteins in Platyhelminthes and Rotifera

ZP-domain proteins represent an ancient and highly conserved family of extracellular glycoproteins characterized by a bipartite architecture of ZP-N and ZP-C subdomains, which mediate polymerization into fibrillar matrices essential for tissue integrity and reproduction across metazoans [3]. While their roles have been extensively characterized in vertebrates and several invertebrate models, direct experimental evidence regarding their specific functions in Platyhelminthes and Rotifera remains notably limited. Comparative genomic analyses of parasitic and free-living flatworms have confirmed the presence of genes encoding canonical ZP-domains, suggesting a deep evolutionary conservation of these structural modules within the phylum [3,40]. Furthermore, recent proteomic investigations have demonstrated that certain platyhelminths secrete extracellular vesicles containing matrix-related glycoproteins exhibiting potential structural homology to vertebrate egg-coat components [41]. Despite these compelling genomic and proteomic indications, direct biochemical or genetic studies validating the precise functional roles of these specific proteins in flatworm reproduction or tissue morphogenesis are currently lacking, and most functional inferences remain computationally derived rather than experimentally verified.

In the case of Rotifera, the situation is even more constrained, with no direct functional studies or specific protein characterizations currently available in the literature. Consequently, our understanding of rotifer ZP proteins relies heavily on inferences drawn from comparative studies of closely related lophotrochozoan taxa [42,43]. For instance, in mollusks such as the abalone Haliotis, ZP-domain proteins like the vitelline envelope receptor for lysin (VERL) are major components of the egg coat and exhibit rapid adaptive evolution under positive selection to mediate species-specific gamete recognition [21,44]. Similarly, recent discoveries in nemerteans have identified novel ZP-domain-containing proteins implicated in fertilization processes [45]. While it is highly probable that rotifers utilize conserved ZP-domain families for egg coat formation and the establishment of reproductive barriers, this remains a computational prediction rather than an experimentally verified fact. The evolutionary plasticity of ZP-domains, reflected in lineage-specific gene duplications and paralog expansions observed in related taxa [3,42], suggests that similar diversification events likely occurred in rotifers, yet the precise molecular identities and functional specializations of these proteins await direct characterization.

The functional versatility of the ZP-domain is further highlighted by studies in model organisms such as the nematode Caenorhabditis elegans, where ZP-domain proteins have been shown to shape tubular organs through their matrix-forming properties, utilizing either the ZP-N or ZP-C subdomains independently [3,46]. This structural flexibility suggests that the ZP-domain proteins in Platyhelminthes and Rotifera could similarly be adapted for diverse extracellular matrix functions beyond simple reproductive envelopes, potentially contributing to embryonic protection, environmental resilience, and tissue organization [3]. However, a significant gap remains in the direct molecular and genetic characterization of individual ZP-domain genes within these specific phyla. Therefore, while strong comparative evidence supports the presence of these matrix-forming glycoproteins in flatworms and rotifers, comprehensive functional assays demonstrating their exact biological roles, particularly in species-specific reproductive interactions, remain an important and unresolved frontier for future research.

### 2.4. Homology and Evolutionary Significance

**Figure 1:**
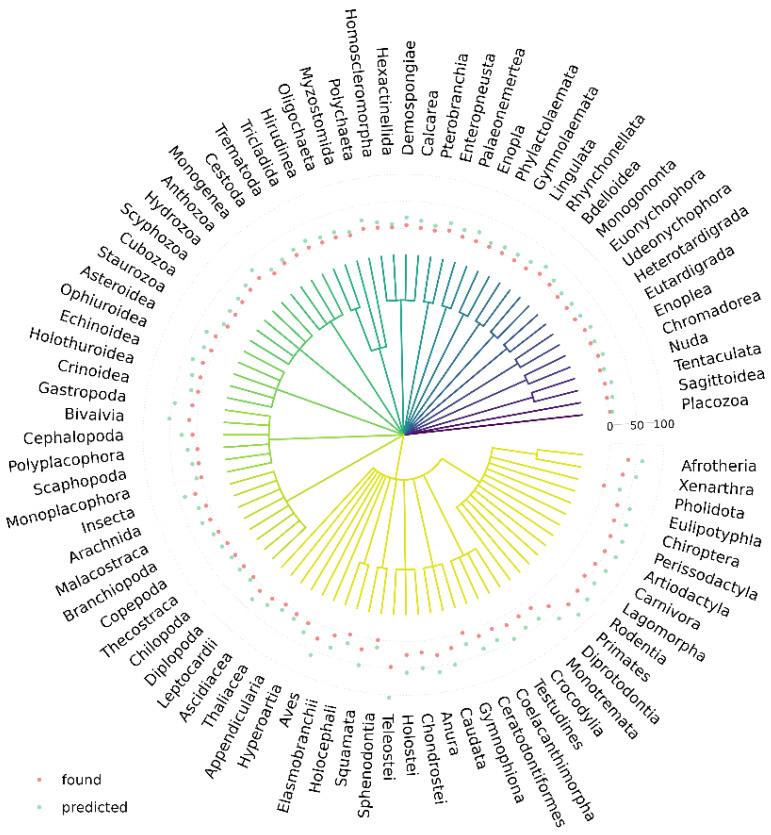
Distribution of ZP-domain proteins across metazoa. Branches colored by major metazoan groups. Red dots indicate experimentally identified (“found”) ZP-domain proteins; green dots indicate computationally predicted occurrences.

The recurring theme across taxa is domain-level deep homology: a shared molecular toolkit underpinning structurally diverse but functionally analogous ECMs (Figure 1). The ZP-domain is a conserved module present in sponges, cnidarians, and vertebrates, illustrating a molecular legacy dating back to the earliest metazoans [14]. Regulatory features such as furin cleavage sites and hydrophobic patches (EHP/IHP) are found in many canonical ZP-domain proteins, but may vary among lineages and protein families [42]. When present, these features likely contribute to controlled polymerization during key reproductive or developmental events [8,39].

Phylogenetic analyses indicate early gene duplications followed by lineage-specific diversification, allowing functional fine-tuning as animal complexity increased [35,47]. Despite vast differences—from sponge cell aggregates to vertebrate egg coats—core structural and functional features remain conserved [48]. Functional roles extend beyond reproduction: in sponges, ZP proteins likely contributed to early cell adhesion; in cnidarians, to embryonic patterning and metamorphosis [14]. This multifunctionality underscores the ZP-domain as a versatile module fundamental to ECM assembly and intercellular communication [37,42].

In conclusion, ZP-domain proteins represent ancient, indispensable components of the metazoan ECM. Their presence in Porifera, Cnidaria, Platyhelminthes, and Rotifera illustrates a continuum of domain-level deep homology, where a conserved molecular framework has been repeatedly adapted for diverse biological functions [3,35,43]. Future integration of genomics, proteomics, and functional genetics in basal metazoans will further illuminate the evolutionary dynamics of this pivotal protein domain [3,8]. A comprehensive overview of the taxonomic distribution, types of evidence, and proposed functions of ZP-domain proteins across major metazoan groups is summarized in Table 1.

## 3. Reproductive ZP-Domain Proteins Across Bilaterian Lineages

ZP-domain proteins form an evolutionarily conserved framework across bilaterian animals, repeatedly co-opted for gamete recognition, sperm binding, and polyspermy prevention. This section examines evidence from Arthropoda, Mollusca, and Chordata to illustrate how divergent evolutionary pressures have remodeled this conserved module.

### 3.1. Arthropoda

In arthropods, the vitelline envelope (VE) and associated egg coverings are specialized extracellular matrices, though their molecular composition varies significantly between lineages. In crustaceans such as the Chinese mitten crab *Eriocheir sinensis*, proteomic analyses indicate that ZP-domain glycoproteins are major components of the seminal fluid and likely contribute to VE assembly, forming a robust filamentous network that provides physical protection and selective sperm access in an aquatic environment [60]. However, in insects, the vitelline membrane is primarily composed of non-ZP proteins such as Vm26A and Vm32E (e.g., in Drosophila melanogaster), which lack the canonical bipartite ZP-N/ZP-C architecture [61,62,63]. Thus, the reliance on ZP-domain proteins for egg-coat formation is not uniform across Arthropoda but rather reflects lineage-specific adaptations to reproductive ecology. Conversely, the vast majority of insects reproduce via internal fertilization, wherein sperm are transferred during copulation, stored for extended periods in the spermatheca, and subsequently released to enter the egg through a specialized micropyle during oviposition [64,65,66]. Consequently, the evolution of the insect egg envelope, including the vitelline envelope and the multi-layered chorion, is fundamentally driven by adaptations to terrestrial life, providing essential protection against desiccation, mechanical stress, and pathogens while maintaining necessary gas exchange [63,67,68,69]. Although the core fold of arthropod VE proteins resembles that of mammalian ZP proteins, they often include additional EGF-like repeats and lineage-specific insertions that reflect their specific reproductive ecologies, ranging from aquatic external fertilization in crustaceans to terrestrial internal fertilization in insects [60,62]. Gene clustering and duplication have facilitated rapid diversification across these lineages, enabling fine-tuned sperm–egg interactions and environmental resilience [61,64]. However, it must be emphasized that no canonical ZP-domain protein with experimentally verified matrix polymerization has been characterized in insects. The presence of ZP-like sequences in insect genomes does not confirm functional equivalence to vertebrate ZP proteins, and the molecular basis of insect vitelline envelope assembly remains largely unknown.

Despite genomic predictions, no functional validation (knockout, RNAi, or heterologous polymerization) of a canonical ZP-domain protein exists for any insect species, leaving the mechanism of vitelline envelope assembly in this lineage unresolved.

### 3.2. Mollusca

In mollusks, the egg envelope is enriched in ZP-like proteins crucial for species-specific fertilization. Proteomic studies detect multiple ZP-domain gene families in reproductive tissues, underscoring their role in constructing protective matrices [70]. In abalones, the interaction between sperm lysin and the egg receptor VERL—mediated by tandem ZP-N repeats—controls gamete recognition [43,70]. Marine mollusks typically have thicker, more complex envelopes than terrestrial species, correlating with environmental demands [43,71]. Transcriptomic data reveal rapid divergence and domain shuffling driven by positive selection, particularly under high-sperm-density conditions [43].

### 3.3. Chordata. From Amphioxus to Vertebrates

The canonical vertebrate zona pellucida emerged as a specialized egg-coat matrix assembled from diversified ZP subfamilies. In invertebrate chordates like amphioxus, the egg coat is simpler and lacks the elaborate ZP structure of vertebrates [42]. Following divergence, gene duplications in the ancestral ZP family gave rise to subfamilies (e.g., ZPAX, ZPB, ZPC), which underwent neofunctionalization [70].

In teleost fish, the egg coat is thin and adapted for external fertilization via a micropyle, supported by a reduced set of ZP proteins [8,70]. In contrast, amphibians and amniotes exhibit increasingly complex coats. Mammals, for instance, possess three to four highly glycosylated ZP proteins that direct species-specific sperm binding and block polyspermy [42,70]. Structural differences reflect the transition from external to internal fertilization, driven by modifications in polymerization and glycosylation [70]. The expansion and specialization of ZP genes have led to remarkable diversity in egg-coat structures across vertebrates [8,70].

The evolution of ZP-domain proteins exemplifies domain-level deep homology and modular evolution across bilaterians. In arthropods, proteins like VMO1 combine conserved folds with lineage-specific domains to meet external fertilization challenges [60,71]. Mollusks demonstrate how the ZP-domain can be elaborated into specialized coatings ensuring reproductive fidelity [43,70,71]. In vertebrates, gene duplication and neofunctionalization led to the complex zona pellucida, tailored to diverse reproductive modes [42,70]. These patterns highlight how evolution repurposes ancient molecular modules to generate novel adaptations. Future interdisciplinary studies will further elucidate how selective pressures shape these critical reproductive molecules, deepening our understanding of the interplay between conserved mechanisms and lineage-specific innovation [43,70].

## 4. Molecular Evolution of ZP Genes

**Figure 2:**
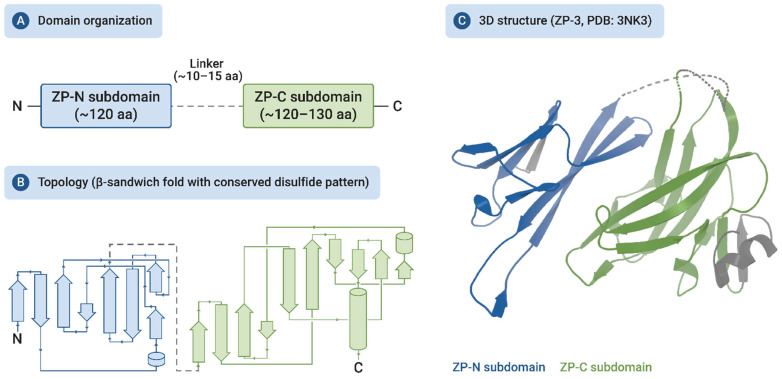
Archetypal architecture and structural organization of the zona pellucida (ZP) domain. (**A**) Domain organization of a canonical ZP-domain-containing protein. The ZP-domain consists of two conserved subdomains, ZP-N (~120 amino acids) and ZP-C (~120–130 amino acids), connected by a short flexible linker (~10–15 amino acids). This bipartite architecture forms the core structural unit responsible for polymerization of ZP proteins into extracellular fibrillar matrices; (**B**) Topology of the ZP-domain (from ZP-3, PDB: 3NK3). Both ZP-N and ZP-C subdomains adopt immunoglobulin-like β-sandwich structures composed predominantly of antiparallel β-strands stabilized by conserved cysteine residues forming intramolecular disulfide bridges; (**C**) Representative three-dimensional structure of the ZP-domain (ZP3; PDB: 3NK3), with ZP-N and ZP-C subdomains shown separately. The spatial arrangement demonstrates the close packing of the two β-sandwich subdomains and their integration into a single functional module.

Previous phylogenetic analyses of ZP-domain-containing proteins across metazoans have revealed a deeply conserved molecular module—hereafter referred to as the canonical ZP-module—that has been repeatedly co-opted for the formation of extracellular egg coats. A critical caveat is that domain-level homology does not imply gene-family orthology. Invertebrate ZP-domain proteins are not orthologous to specific vertebrate ZP subfamilies (ZP1–ZP4), and phylogenetic reconstructions that force 1:1 correspondence across bilaterians are likely overinterpreted. This canonical module is distinct from ZP-like domains found in choanoflagellates, which lack the full bipartite architecture and invariant cysteine pattern. The canonical ZP-domain, composed of ZP-N and ZP-C subdomains, is present in lineages ranging from basal deuterostomes to derived chordates, indicating an ancient evolutionary origin [2] (Figure 2). Studies encompassing 97 metazoan species have identified up to eight distinct ZP subfamilies—ZP1, ZP2, ZP3, ZP4, ZPD, ZPAX, ZPX, and ZPY—whose diversification traces back to early gene duplication events in the vertebrate ancestor [2]. Maximum likelihood phylogenies, supported by synteny analyses, delineate well-defined clades that reflect both domain-level deep homology and lineage-specific expansions [2].

Despite low overall sequence identity, the core architecture of the ZP-domain—characterized by a conserved β-sandwich fold and a pattern of typically conserved cysteine residues forming four intramolecular disulfide bonds—is remarkably preserved [23] (Figure 2B,C). This structural conservation, particularly in the ZP-N and ZP-C subdomains, provides compelling evidence for a shared evolutionary origin across metazoans [2]. In invertebrates such as mollusks and cephalochordates, independent gene duplications have led to lineage-specific domain expansions. For example, the abalone Haliotis vitelline envelope receptor for lysin (VERL) exhibits extensive tandem duplication of ZP-N repeats, functionally analogous to the diversification of vertebrate ZP proteins [2]. These findings underscore that while gene families undergo dynamic evolution in copy number and sequence, the ZP-domain itself remains a conserved evolutionary unit (Figure 2).

The ZP-domain is a deeply conserved structural module present in all major animal lineages, from Porifera and Cnidaria to Chordata [2]. While absent in fungi and plants, its evolutionary origins likely predate the emergence of Metazoa, as ZP-like domains have also been identified in certain unicellular holozoans [72]. Its structural stability, conferred by the conserved disulfide bond network, enables reliable folding under diverse physiological conditions, making it a robust module for extracellular matrix assembly [2,73]. Functionally, ZP-domain proteins mediate species-specific sperm recognition, prevent polyspermy, and provide mechanical protection to the oocyte and embryo. This dual capacity—structural conservation coupled with functional plasticity—exemplifies modular evolution, where a single domain is repurposed across taxa to meet diverse reproductive demands.

The diversification of ZP gene families has been driven by several genetic mechanisms. Gene duplication has been pivotal, with ancestral ZP-like genes likely undergoing multiple duplications to give rise to paralogous lineages that acquired specialized roles in egg-coat assembly and gamete interaction. Exon shuffling and domain recombination have further contributed to innovation, incorporating auxiliary domains such as trefoil motifs or LDL-receptor domains that modulate protein interactions and enhance functional specificity [17].

As demonstrated in previous studies, positive Darwinian selection acts on regions involved in gamete recognition, particularly in sperm-binding interfaces of ZP2 and ZP3, where elevated dN/dS ratios indicate adaptive evolution driven by sexual conflict and sperm competition [17]. Maximum likelihood frameworks across mammals, including rodents and primates, identified specific codons under selection, with rigorous controls excluding artifacts like misalignment or recombination [15,16,17]. Conversely, lineage-specific loss shaped ZP genes (e.g., ZP4 pseudogene in mice, ZPAX nonfunctional in humans), reflecting shifts in reproductive strategies [2]. Avian ZPB and invertebrate VERL show functional divergence and neofunctionalization, enabling species-specific recognition [2]. These cases illustrate how conserved modules support rapid evolution, reproductive isolation, and speciation.

In summary, the molecular evolution of ZP genes exemplifies modular evolution in metazoans. The conserved ZP-N/ZP-C architecture has been maintained since the dawn of animal evolution, serving as a synapomorphy for extracellular matrix formation [2]. Gene duplication, domain shuffling, positive selection, and gene loss have collectively shaped a dynamic evolutionary landscape, allowing a single domain to support a wide array of reproductive strategies [17]. This interplay of stability and adaptability not only underpins reproductive isolation and speciation but also reflects broader principles of developmental module evolution [2]. Future integration of structural biology, comparative genomics, and functional assays will further elucidate the evolutionary forces shaping ZP gene diversity [23].

## 5. Functional Diversification and Co-Option of the Zona Pellucida Across Lineages

In basal metazoans, extracellular matrices containing ZP-like proteins functioned as protective barriers, shielding oocytes from mechanical stress, pathogens, and polyspermy [1]. Rather than representing a linear progression from a “primitive” passive defense to an “advanced” recognition interface, the evolutionary history of these proteins is better understood through the lens of repeated co-option and lineage-specific functional diversification. Across diverse taxa, varying selective pressures associated with distinct reproductive ecologies have repeatedly recruited these conserved structural modules for specialized roles. For instance, in certain vertebrate lineages, this diversification facilitated the emergence of specific receptor–ligand interactions mediating precise sperm recognition [4], whereas in other lineages, ZP-domains were co-opted for alternative extracellular matrix functions. This modular versatility highlights how a conserved structural framework can be differentially adapted to meet a wide array of lineage-specific reproductive demands.

Structural refinements—such as enhanced glycosylation and regulated polymerization—enabled the presentation of discrete binding epitopes on key proteins like ZP2 and ZP3, facilitating signal transduction upon sperm contact [4,74]. Comparative analyses across taxa reveal a clear trend: increasing complexity in gamete recognition systems correlates with progressive molecular specialization of the ZP, reflecting a shift from generalized protection to active mediation of fertilization.

A central role of the ZP in reproductive isolation is evident in the rapid adaptive evolution of sperm-binding domains. In murine rodents, for example, nucleotide divergence in the sperm-interaction region of ZP3 is driven by positive selection, reinforcing species-specific fertilization and minimizing hybridization [75]. Similarly, in abalone, ZP-domain proteins such as the vitelline envelope receptor for lysin (VERL) exhibit accelerated divergence, preventing interspecific fertilization and maintaining reproductive boundaries [76]. Transgenic studies further confirm this specificity; human sperm bind exclusively to humanized ZP2, underscoring the molecular precision of gamete recognition [77]. Oviductal glycoproteins also contribute to species specificity, highlighting a multi-layered regulatory system that fine-tunes reproductive compatibility [4,78].

The coevolution of ZP components and sperm ligands exemplifies a dynamic molecular arms race. While ZP3 has traditionally been highlighted for its interaction with sperm surface receptors (e.g., SED1, GalT, sp56) to initiate binding and the acrosome reaction [79,80], the contemporary model of sperm–egg recognition is more complex. Recent evidence emphasizes the critical role of the ZP2 N-terminal region, which serves as a major secondary binding site essential for maintaining sperm attachment post-acrosome reaction [33,77]. Furthermore, recognition is not mediated exclusively by carbohydrate moieties; protein-backbone determinants are now recognized as crucial contributors to species-specific binding specificity [77,79]. This intricate recognition system is further modulated by oviductal glycoproteins (e.g., oviductin), which coat the egg and fine-tune the glycosylation patterns and structural presentation of ZP proteins, adding a vital layer of species-specific regulation [4,78]. Together, these combined glycan and protein-backbone determinants act as sophisticated molecular barcodes to prevent heterospecific fertilization [30,55]. Nevertheless, the relative contribution of glycans versus protein backbone to species-specific recognition remains unresolved and likely varies across taxa. In some lineages (e.g., abalone), protein–protein interactions dominate, whereas in mammals, evidence supports a dual or context-dependent model. The rapid evolution of these domains under positive Darwinian selection highlights their role in reproductive fitness and has significant implications for understanding infertility and improving assisted reproductive technologies [27,30].

Subtle sequence variations in N-terminal domains of ZP proteins can profoundly influence binding specificity. In abalone, VERL and its paralog VEZP14 undergo rapid adaptive divergence under positive selection, coevolving with sperm lysin to ensure species-specific recognition [76,81]. Similarly, post-fertilization cleavage of the N-terminal region of ZP2 by ovastacin alters the egg coat’s architecture, blocking polyspermy and reinforcing monospermic fertilization [33]. These mechanisms underscore the functional significance of precise molecular interactions in reproductive success. Genomic studies reveal correlated rates of amino acid substitution and gene family expansions in gamete recognition proteins, indicating that sexual selection, sperm competition, and host–pathogen dynamics drive sustained adaptive evolution [73]. The coevolution of ZP proteins and their sperm ligands forms a critical axis of reproductive isolation, promoting speciation across diverse metazoan lineages [73].

The zona pellucida transformed from a simple protective coat into a refined recognition interface. Passive defense functions gave way to molecular add-ons—glycosylation, domain shuffling, proteolytic control—that ensure precise sperm binding and reproductive isolation. Positively selected sperm-interaction domains fuel rapid divergence, reinforcing species boundaries via coevolutionary conflict. This interplay between ZP and sperm proteins illustrates how sexual selection and interspecific competition shape reproductive diversification across animals [1,4].

### Evolutionary Scenarios for the Origin of the Zona Pellucida

Recent comparative genomic and proteomic studies challenge the traditional view that the zona pellucida is a vertebrate-specific innovation. Instead, evidence indicates that ZP-domain-containing proteins are present in early-branching metazoans such as Porifera (sponges) and Placozoa, where they function in non-reproductive contexts [72]. Analyses of the sponge mesohyl and cnidarian basal lamina reveal that ZP-domain proteins are integral components of the extracellular matrix (ECM), contributing to structural support and cell adhesion [43]. Genomic data further demonstrate that key ZP-domain genes are present in pre-bilaterian lineages, suggesting that these domains were co-opted for gamete recognition and egg-coat formation during later evolutionary transitions [72].

In basal metazoans, the ancestral role of these ECM proteins was likely the maintenance of tissue integrity and facilitation of intercellular adhesion—functions that laid the molecular groundwork for their subsequent adaptation in reproductive systems [72,82]. This supports the hypothesis that the ZP evolved from ancient ECM components that were repurposed in lineages with external development, where a protective egg coat provides a selective advantage during fertilization [82].

**Figure 3:**
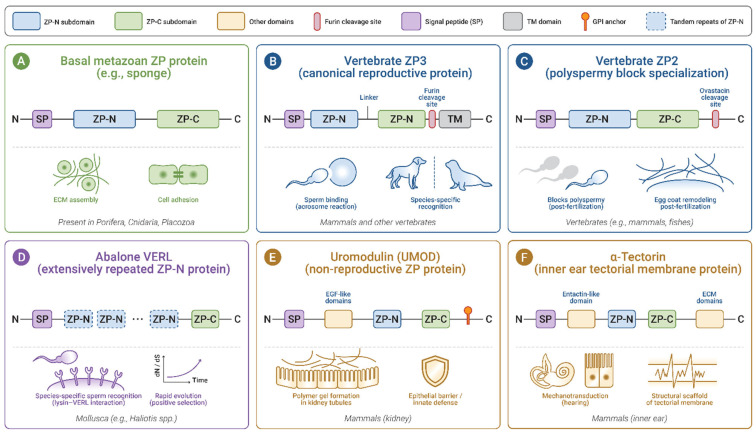
Structural and functional diversity of ZP-domain proteins. (**A**) Basal metazoan ZP protein: simple ZP-N–ZP-C architecture; functions in ECM assembly and cell adhesion; (**B**) Vertebrate ZP3: includes signal peptide, ZP module, and cleavage site; mediates sperm binding and species-specific recognition; (**C**) Vertebrate ZP2: ZP-domain with ovastacin cleavage site; involved in polyspermy block and post-fertilization remodeling; (**D**) Abalone VERL: multiple tandem ZP-N repeats followed by ZP-C; mediates lysin-dependent sperm recognition and shows rapid evolution; (**E**) Uromodulin (UMOD): ZP-domain with additional domains and GPI anchor; forms polymeric filaments in kidney tubules and contributes to epithelial defense; (**F**) α-Tectorin: ZP-domain with additional ECM domains; functions in mechanotransduction and structural organization of the tectorial membrane.

The ZP-domain itself is a highly conserved module composed of two subdomains—ZP-N and ZP-C—characterized by conserved disulfide bonding patterns and the capacity to form polymeric fibrillar networks. A consensus furin cleavage site is a feature of many canonical ZP-domain proteins, although it is not universally present across all ZP-domain-containing proteins [43] (Figure 2A,B). This intrinsic modularity has enabled the repeated co-option of the ZP-domain across diverse biological structures. In mammals, ZP-domain proteins are not restricted to reproductive tissues; they are also found in the tectorial membrane of the inner ear and in the kidney glycoprotein uromodulin UMOD (Figure 3E,F). In reptiles, ZP-domain proteins contribute to egg capsule formation, illustrating convergent co-option in which similar molecular modules are independently recruited for analogous functions in distantly related lineages.

Evolutionary “domain shuffling”—via exon recombination and gene fusion—has further diversified ZP-domain proteins, generating novel architectures in which the ZP-domain is combined with other functional motifs (e.g., trefoil or EGF-like domains) [17]. This modular versatility extends ECM functionality from basic structural assembly to specialized roles in gamete recognition and signaling, reinforcing the ZP-domain’s status as a fundamental building block in the metazoan extracellular toolkit [17].

Phylogenetic analyses suggest that ZP-domain proteins diverged from an ancestral set of adhesion and differentiation-associated modules. Homologs involved in cell adhesion, tissue morphogenesis, and epithelial differentiation—such as netrins and collagen-like ECM proteins—exhibit structural features reminiscent of the ZP-domain [72,82]. Notably, ZP-like domains have been identified in unicellular holozoans such as *Salpingoeca rosetta*, where they are implicated in colony formation and cellular cohesion—processes foundational to the evolution of multicellularity [72].

The evolution of the ECM represents a transition from general structural roles to specialized functions, with the ZP exemplifying this trajectory through gene co-option and neofunctionalization [6,7]. Core ECM components, conserved across metazoans, reflect a shared evolutionary heritage [72,83]. The structural conservation of the ZP-domain, coupled with adaptive diversification in reproductive contexts, highlights the balance between maintaining essential ECM functions and evolving new roles in fertilization and species isolation [7].

Molecular, genomic, and proteomic data trace the ZP back to ancient ECM proteins in basal metazoans. Modularity of the ZP-domain—ZP-N and ZP-C subdomains—and its reshuffling into new genomic contexts drove convergent evolution of specialized egg coats across separate lineages [17]. Adhesion and differentiation factors originally serving tissue integrity were co-opted for gamete recognition and embryo protection, marking a major functional shift in reproductive biology [72].

## 6. Conclusions

The zona pellucida and its structural analogs represent a remarkable example of evolutionary innovation built upon deep molecular conservation. Far from being a vertebrate-specific novelty, the ZP-domain constitutes an ancient metazoan module whose origins predate the divergence of major animal lineages. Evidence from basal metazoans, including Porifera, Cnidaria, and Placozoa, demonstrates that ZP-domain proteins originally functioned in general extracellular matrix (ECM) organization, mediating cell adhesion and tissue integrity. Over evolutionary time, this conserved structural framework was repeatedly co-opted for specialized reproductive roles, particularly in the formation of protective egg coats and the mediation of species-specific gamete recognition.

The core architecture of the ZP-domain, comprising ZP-N and ZP-C subdomains, conserved disulfide bonding patterns, and regulatory motifs such as the furin cleavage site, is preserved across phylogenetically distant taxa. This structural stability enables the formation of polymeric, fibrillar matrices essential for oocyte protection and fertilization. However, this conservation is counterbalanced by extensive sequence divergence and lineage-specific domain shuffling, driven by positive selection, gene duplication, and neofunctionalization. These processes have fine-tuned ZP proteins to meet diverse reproductive strategies, ranging from external fertilization in aquatic invertebrates to internal fertilization in mammals.

The accumulated evidence supports several robust conclusions. First, the bipartite ZP-N/ZP-C module, rather than merely individual ZP-like sequences, is a metazoan synapomorphy present from Porifera to vertebrates and functions as a conserved polymerization unit for ECM assembly. Second, this module has been independently co-opted for reproductive functions, especially egg-coat formation, across multiple lineages. Third, positive selection consistently targets sperm-interaction interfaces, including ZP2, ZP3, and VERL, thereby driving reproductive isolation and reinforcing species boundaries. Fourth, post-fertilization modifications, such as ovastacin-mediated cleavage of ZP2, represent a conserved mechanism for polyspermy block, at least within deuterostomes.

A key evolutionary trend is the functional shift from a passive protective barrier to an active, signaling-competent interface for sperm–egg interaction. In vertebrates and many invertebrates, ZP glycoproteins have evolved to serve as ligands for sperm receptors, with binding regions undergoing rapid adaptive evolution. This coevolutionary arms race, exemplified by abalone lysin–VERL or mammalian ZP3–sperm ligand interactions, reinforces reproductive isolation and contributes to speciation. Post-translational modifications, particularly glycosylation, further enhance functional specificity by acting as molecular barcodes that ensure species-restricted fertilization. Moreover, the modular nature of the ZP-domain has enabled its recruitment beyond the reproductive system, as seen in the tectorial membrane of the inner ear and the kidney glycoprotein uromodulin. This widespread reuse underscores the domain’s versatility as a fundamental building block in metazoan extracellular biology.

Despite these advances, major uncertainties remain. One critical issue concerns orthology versus domain homology; vertebrate ZP genes, including ZP1 through ZP4, ZPAX, and ZPD, do not have clear one-to-one orthologs in most invertebrates despite sharing the same ZP-domain. Whether this reflects deep divergence, convergent evolution of the domain, or extensive lineage-specific gene loss remains unknown. A second unresolved question relates to the relative contribution of glycans versus protein backbone in species-specific sperm recognition. While glycosylation is clearly important for sperm binding in mammals, conflicting evidence across taxa leaves the relative roles of glycan determinants and protein–protein interactions uncertain. Third, insects present a paradox; no canonical ZP-domain protein with the full bipartite architecture and experimentally validated polymerization function has been characterized in insect egg coats. Existing candidates, such as VMO1, lack direct evidence for ZP-dependent matrix assembly, and it remains an open question whether insects lost canonical ZP polymers or employ unrelated mechanisms. Fourth, a broader functional validation gap exists: almost all conclusions regarding ZP-domain proteins in non-model invertebrates, including Platyhelminthes, Rotifera, and most mollusks, rest on computational predictions rather than on knockout, RNA interference, or protein localization data.

Resolving these gaps will require targeted methodological innovation. Glycoproteomics, specifically site-specific glycan mapping on native ZP proteins from multiple species, could distinguish glycan-driven from protein-driven recognition. Single-cell ovarian transcriptomics will reveal which cell types express which ZP paralogs, particularly in non-model taxa. CRISPR/Cas9 or RNA interference in emerging model organisms, such as Nematostella, Branchiostoma, or beetles, could directly test polymerization and sperm-binding functions in vivo. Protein localization by immuno-electron microscopy in understudied lineages, including rotifers, flatworms, and crustaceans, would distinguish true ZP matrices from predictions based solely on homology. Finally, codon-level selection analyses, employing dN/dS with site and branch-site models, applied to newly assembled genomes from phylogenetically critical taxa such as placozoans and xenacoelomorphs could reveal when and where positive selection on ZP-domains intensified.

In summary, the evolutionary history of ZP proteins illustrates a central principle in molecular evolution: the repurposing of conserved structural modules for novel biological functions. The transition from general ECM components to specialized gamete recognition molecules exemplifies how developmental and reproductive systems evolve, reflecting the interplay of domain-level deep homology and adaptive innovation. An integrated comparative framework combining structural biology, comparative genomics, and functional assays will not only transform ZP-domain biology from a descriptive account of deep homology into a mechanistic understanding of reproductive evolution but also hold significant implications for understanding infertility, developing non-hormonal contraceptives, and elucidating broader principles of reproductive evolution.

## Figures and Tables

**Table 1:** Distribution and functional characterization of ZP-domain proteins across Metazoa.

Major Taxonomic Group	Representative ZP-Domain Protein(s)	Representative Species	Type of Evidence	Proposed/Confirmed Function	Key References
**Porifera**	ZP-domain ECM proteins (putative)	*Amphimedon queenslandica* and other sponges	Genomic, transcriptomic prediction of ZP-domains in ECM surveys	ECM assembly and structural scaffold in early metazoan extracellular matrices	[49]
**Cnidaria**	Mesoglein (ZP-domain ECM protein)	*Aurelia aurita*	Biochemical, immunocytochemistry, sequence domain analysis	Major mesoglea structural component; forms elastic fibers; plate/contact plate at oocyte–epithelium interface, putative ZP precursor	[36,37]
	ZP-domain matrisome proteins (multiple)	*Nematostella vectensis*	Bioinformatic prediction, proteomics of decellularized mesoglea	Complex ECM assembly and remodeling across life stages; early diversification of ZP-containing ECM proteins	[50]
**Platyhelminthes**	ZP-domain proteins (predicted)	Parasitic and free-living flatworms	Phylogenomic/bioinformatic surveys of ZP-domains	ZP-domains in apical extracellular matrices, morphogenesis, protective layers; diversification and disulfide reshuffling	[51]
**Rotifera**	ZP-domain proteins (predicted)	Lophotrochozoan/rotifer representatives	InterProScan/Pfam ZP-domain screens	Egg coat/ECM-related matrix assembly roles inferred from ZP architecture	[43]
**Ecdysozoa (Nematoda model)**	LET-653 (ZP protein), FBN-1, cuticlin ZP-domains	*Caenorhabditis elegans*	Genetic loss-of-function, localization, structural modeling	ZP-domain proteins in apical ECM, tube shaping, epidermal resistance to deformation; ZPc-mediated matrix incorporation	[46,51]
**Mollusca (Gastropoda)**	VERL and multiple VE ZP-proteins	*Haliotis* spp. (abalone)	Proteomics of VE, ESTs, molecular evolution	Major vitelline envelope (VE) structural proteins; rapidly evolving repeats implicated in species-specific sperm binding and reproductive isolation	[5,21,52]
**Mollusca (Bivalvia, Limpets)**	EGFZP, EGFL (shell matrix ZP-proteins)	*Pinctada fucata*, *Nipponacmea fuscoviridis*, *Lottia gigantea*	Genomic, transcriptomic, expression, protein–protein interaction assays	ZP-domain mediates shell matrix assembly; interaction hub for multiple shell matrix proteins; roles in calcitic and aragonitic shell formation	[43]
**Cephalochordata**	BbZP1–BbZP5	*Branchiostoma belcheri*	Proteomics of egg coat, FISH, immunohistochemistry, phylogenetics	Principal egg-coat (EC) components; ECM polymerization; ancestral chordate ZP egg-coat system	[8]
**Vertebrata (Teleosts, Amphibians, Birds)**	ZP1, ZP2, ZP3/ZPC, ZPAX, ZPD, additional VE ZPs	*Oryzias latipes*, *Xenopus*, *Gallus*	Genomic, expression, structural, phylogenetic analyses	Egg coat/VE assembly, protection, sperm binding (subset, esp. ZP3/ZPC), polyspermy block; lineage-specific expansions	[8,42,52,53,54]
**Vertebrata (Mammals)**	ZP1–ZP4	*Mus musculus*, *Bos taurus*, *Homo sapiens*	Knockouts, structural biology, recombinant binding, functional assays	Structural zona pellucida matrix, species-restricted sperm binding (ZP2/ZP3/ZP4, ZP-N repeats), post-fertilization hardening and polyspermy block	[42,52,53,55,56,57,58,59]
**Vertebrata (Non-reproductive ZP-proteins)**	UMOD, TECTA, CUZD1 and other ZP-domain proteins	*Homo sapiens*, diverse vertebrates	Genomic surveys, structural and evolutionary analyses	ZP-domains in non-reproductive ECMs (e.g., kidney, inner ear); conserved polymerization with diversified functions	[23,51,58]

Abbreviations: ECM, extracellular matrix; VE, vitelline envelope; ZP, zona pellucida; VERL, vitelline envelope receptor for lysin; EGFZP, EGF-like and ZP-domain–containing protein; EGFL, EGF-like protein; ZP-N/ZPc, N-terminal and C-terminal subdomains of the ZP module.

## Data Availability

No new data were created or analyzed in this study. Data sharing is not applicable to this article.

## References

[1] Claw K.G., Swanson W.J. (2012). Evolution of the egg: New findings and challenges. Annu. Rev. Genom. Hum. Genet..

[2] Feng J.-M., Tian H.-F., Hu Q.-M., Meng Y., Xiao H.-B. (2018). Evolution and multiple origins of zona pellucida genes in vertebrates. Biol. Open.

[3] Litscher E.S., Wassarman P.M. (2020). Zona pellucida proteins, fibrils, and matrix. Annu. Rev. Biochem..

[4] Moros-Nicolás C., Chevret P., Jiménez-Movilla M., Algarra B., Cots-Rodríguez P., González-Brusi L., Avilés M., Izquierdo-Rico M.J. (2021). New insights into the mammalian egg zona pellucida. Int. J. Mol. Sci..

[5] Wilburn D.B., Swanson W.J. (2017). The “ZP-domain” is not one, but likely two independent domains. Mol. Reprod. Dev..

[6] Hasegawa A., Shibahara H., Hasegawa A. (2022). Structure of ZP. Gamete Immunology.

[7] Killingbeck E.E., Swanson W.J. (2018). Egg coat proteins across metazoan evolution. Curr. Top. Dev. Biol..

[8] Xu Q., Li G., Cao L., Wang Z., Ye H., Chen X., Yang X., Wang Y., Chen L. (2012). Proteomic characterization and evolutionary analyses of zona pellucida domain-containing proteins in the egg coat of the cephalochordate, *Branchiostoma belcheri*. BMC Evol. Biol..

[9] Wu T., Cheng Y., Liu Z., Tao W., Zheng S., Wang D. (2018). Bioinformatic analyses of zona pellucida genes in vertebrates and their expression in Nile tilapia. Fish. Physiol. Biochem..

[10] Sano K., Shimada S., Mibu H., Taguchi M., Ohsawa T., Kawaguchi M., Yasumasu S. (2022). Lineage-specific evolution of zona pellucida genes in fish. J. Exp. Zool. Part B Mol. Dev. Evol..

[11] Glasauer S.M.K., Neuhauss S.C.F. (2014). Whole-genome duplication in teleost fishes and its evolutionary consequences. Mol. Genet. Genom. MGG.

[12] Guo B., Wagner A., He S., Friedberg F. (2011). Duplicated gene evolution following whole-genome duplication in teleost fish. Gene Duplication.

[13] Ozernyuk N.D., Myuge N.S. (2013). Large-scale genome duplications and paralog divergence in fish. Russ. J. Genet..

[14] Bokhove M., Jovine L. (2018). Structure of zona pellucida module proteins. Curr. Top. Dev. Biol..

[15] Ramm S.A., Oliver P.L., Ponting C.P., Stockley P., Emes R.D. (2008). Sexual selection and the adaptive evolution of mammalian ejaculate proteins. Mol. Biol. Evol..

[16] Swanson W.J., Nielsen R., Yang Q. (2003). Pervasive adaptive evolution in mammalian fertilization proteins. Mol. Biol. Evol..

[17] Rivera A.M., Swanson W.J. (2022). The importance of gene duplication and domain repeat expansion for the function and evolution of fertilization proteins. Front. Cell Dev. Biol..

[18] Clark N.L. (2008). Adaptive evolution of primate sperm proteins. Encyclopedia of Life Sciences.

[19] Gasper J., Swanson W.J. (2006). Molecular population genetics of the gene encoding the human fertilization protein zonadhesin reveals rapid adaptive evolution. Am. J. Hum. Genet..

[20] González D.P., Lamb H.V., Partida D., Wilson Z.T., Harrison M.C., Prieto J.A., Moresco J.J., Diedrich J.K., Yates J.R., Olson S.K. (2018). CBD-1 organizes two independent complexes required for eggshell vitelline layer formation and egg activation in *C. elegans*. Dev. Biol..

[21] Aagaard J.E., Yi X., MacCoss M.J., Swanson W.J. (2006). Rapidly evolving zona pellucida domain proteins are a major component of the vitelline envelope of abalone eggs. Proc. Natl. Acad. Sci. USA.

[22] Morgan C.C., Hart M.W. (2019). Molecular evolution of mammalian genes with epistatic interactions in fertilization. BMC Evol. Biol..

[23] Rivera A.M., Wilburn D.B., Swanson W.J. (2022). Domain expansion and functional diversification in vertebrate reproductive proteins. Mol. Biol. Evol..

[24] Yonezawa N. (2014). Posttranslational modifications of zona pellucida proteins. Adv. Exp. Med. Biol..

[25] Sinowatz F., Töpfer-Petersen E., Kölle S., Palma G. (2001). Functional morphology of the zona pellucida. Anat. Histol. Embryol..

[26] Clark G.F. (2010). The mammalian zona pellucida: A matrix that mediates both gamete binding and immune recognition?. Syst. Biol. Reprod. Med..

[27] Wassarman P.M., Litscher E.S., Skinner M.K. (2018). The zona pellucida. Encyclopedia of Reproduction.

[28] Zhou Z., Ni C., Wu L., Chen B., Xu Y., Zhang Z., Mu J., Li B., Yan Z., Fu J. (2019). Novel mutations in ZP1, ZP2, and ZP3 cause female infertility due to abnormal zona pellucida formation. Hum. Genet..

[29] Gupta S.K. (2023). Zona pellucida glycoproteins: Relevance in fertility and development of contraceptive vaccines. Am. J. Reprod. Immunol..

[30] Serres C., Auer J., Petit F., Patrat C., Jouannet P. (2008). Molecules involved in sperm–zona pellucida interaction in mammals. Role in human fertility. J. Soc. Biol..

[31] Hasegawa A., Fukui A., Shibahara H. (2017). The current perspectives on the mammalian zona pellucida. J. Mamm. Ova Res..

[32] Wodak S.J., Velankar S. (2023). Structural biology: The transformational era. Proteomics.

[33] Nishio S., Emori C., Wiseman B., Fahrenkamp D., Dioguardi E., Zamora-Caballero S., Bokhove M., Han L., Stsiapanava A., Algarra B. (2024). ZP2 cleavage blocks polyspermy by modulating the architecture of the egg coat. Cell.

[34] Doerr S., Zhou P., Ragkousi K. (2024). Origin and development of primary animal epithelia. BioEssays News Rev. Mol. Cell. Dev. Biol..

[35] Conci N., Wörheide G., Vargas S. (2019). New non-bilaterian transcriptomes provide novel insights into the evolution of coral skeletomes. Genome Biol. Evol..

[36] Matveev I.V., Adonin L.S., Shaposhnikova T.G., Podgornaya O.I. (2012). *Aurelia aurita*—Cnidarian with a prominent medusoid stage. J. Exp. Zool. Part B Mol. Dev. Evol..

[37] Adonin L.S., Podgornaya O.I., Matveev I.V., Shaposhnikova T.G. (2009). Plate in the zone of oocyte and germinal epithelium contact in Scyphomedusa *Aurelia aurita* binds antibodies to ZP-domain protein mesoglein. Cell Tissue Biol..

[38] Adonin L.S., Shaposhnikova T.G., Podgornaya O. (2012). *Aurelia aurita* (Cnidaria) oocytes’ contact plate structure and development. PLoS ONE.

[39] Levitan S., Sher N., Brekhman V., Ziv T., Lubzens E., Lotan T. (2015). The making of an embryo in a basal metazoan: Proteomic analysis in the sea anemone *Nematostella vectensis*. Proteomics.

[40] International Helminth Genomes Consortium (2019). Comparative genomics of the major parasitic worms. Nat. Genet..

[41] Ancarola M.E., Maldonado L.L., García L.C.A., Franchini G.R., Mourglia-Ettlin G., Kamenetzky L., Cucher M.A. (2023). A Comparative Analysis of the Protein Cargo of Extracellular Vesicles from Helminth Parasites. Life.

[42] Litscher E.S., Wassarman P.M. (2014). Evolution, structure, and synthesis of vertebrate egg-coat proteins. Trends Dev. Biol..

[43] Shimizu K., Takeuchi T., Negishi L., Kurumizaka H., Kuriyama I., Endo K., Suzuki M. (2022). Evolution of epidermal growth factor (EGF)-like and zona pellucida domains containing shell matrix proteins in mollusks. Mol. Biol. Evol..

[44] Clark N.L., Gasper J., Sekino M., Springer S.A., Aquadro C.F., Swanson W.J. (2009). Coevolution of interacting fertilization proteins. PLoS Genet..

[45] Ikenaga J., Yoshida K., Yoshida M. (2024). Identification of Six Novel Proteins Containing a ZP Module from Nemertean Species. Biomolecules.

[46] Cohen J.D., Bermudez J.G., Good M.C., Sundaram M. (2020). A *C. elegans* Zona Pellucida domain protein functions via its ZPc domain. PLoS Genet..

[47] Dzik J.M. (2010). The ancestry and cumulative evolution of immune reactions. Acta Biochim. Pol..

[48] Wassarman P.M., Litscher E.S. (2022). Female fertility and the zona pellucida. eLife.

[49] Draper G.W., Shoemark D., Adams J.C. (2019). Modelling the early evolution of extracellular matrix from modern Ctenophores and Sponges. Essays Biochem..

[50] Bergheim B.G., Cole A.G., Rettel M., Stein F., Redl S., Hess M.W., Ikmi A., Özbek S. (2025). Molecular dynamics of the matrisome across sea anemone life history. eLife.

[51] Weadick C.J. (2020). Molecular Evolutionary Analysis of Nematode Zona Pellucida (ZP) Modules Reveals Disulfide-Bond Reshuffling and Standalone ZP-C Domains. Genome Biol. Evol..

[52] Monné M., Jovine L. (2011). A Structural View of Egg Coat Architecture and Function in Fertilization. Biol. Reprod..

[53] Monné M., Han L., Schwend T., Burendahl S., Jovine L. (2008). Crystal structure of the ZP-N domain of ZP3 reveals the core fold of animal egg coats. Nature.

[54] Callebaut I., Mornon J., Monget P. (2007). Isolated ZP-N domains constitute the N-terminal extensions of Zona Pellucida proteins. Bioinformatics.

[55] Tumova L., Zigo M., Sutovsky P., Sedmikova M., Postlerova P. (2021). Ligands and receptors involved in the sperm–zona pellucida interactions in mammals. Cells.

[56] Dilimulati K., Orita M., Undram G., Yonezawa N. (2021). Sperm-binding regions on bovine egg zona pellucida glycoprotein ZP4 studied in a solid supported form on plastic plate. PLoS ONE.

[57] Dilimulati K., Orita M., Yonahara Y., Imai F.L., Yonezawa N. (2022). Identification of Sperm-Binding Sites in the N-Terminal Domain of Bovine Egg Coat Glycoprotein ZP4. Int. J. Mol. Sci..

[58] Jovine L., Darie C.C., Litscher E., Wassarman P.M. (2005). Zona pellucida domain proteins. Annu. Rev. Biochem..

[59] Monné M., Han L., Jovine L. (2006). Tracking Down the ZP Domain: From the Mammalian Zona Pellucida to the Molluscan Vitelline Envelope. Semin. Reprod. Med..

[60] He L., Li Q., Liu L., Wang Y., Xie J., Yang H., Wang Q. (2015). A catalog of proteins expressed in the AG secreted fluid during the mature phase of the Chinese mitten crabs (*Eriocheir sinensis*). PLoS ONE.

[61] Jagadeeshan S., Singh R.S. (2007). Rapid evolution of outer egg membrane proteins in the *Drosophila melanogaster* subgroup: A case of ecologically driven evolution of female reproductive traits. Mol. Biol. Evol..

[62] Mazzini M., Callaini G., Mencarelli C. (1984). A comparative analysis of the evolution of the egg envelopes and the origin of the yolk. Ital. J. Zool..

[63] Rezende G.L., Vargas H.C.M., Moussian B., Cohen E. (2016). Composite Eggshell Matrices: Chorionic Layers and Sub-chorionic Cuticular Envelopes. The Insect Egg.

[64] Pascini T.V., Martins G. (2017). The insect spermatheca: An overview. Zoology.

[65] Pascini T.V., Ramalho-Ortigão M., Ribeiro J., Jacobs-Lorena M., Martins G. (2020). Transcriptional profiling and physiological roles of *Aedes aegypti* spermathecal-related genes. BMC Genom..

[66] Yamada H., Hood-Nowotny R., Resch C., Bouyer J., Gruber R., Oliva C. (2024). Sperm Storage and Use Following Multiple Insemination in *Aedes albopictus*: Encouraging Insights for the Sterile Insect Technique. Insects.

[67] Li T.-H., Wang X., Desneux N., Wang S., Zang L. (2024). Egg coverings in insects: Ecological adaptation to abiotic and biotic selective pressures. Biol. Rev..

[68] Wheeler D. (2009). Chapter 84—Egg Coverings. Encyclopedia of Insects.

[69] Witaliński W. (1987). Egg-Shells in mites: Cytological aspects of vitelline envelope and chorion formation in *Pergamasus barbarus* berlese (Gamasida, Pergamasidae). Int. J. Acarol..

[70] Killingbeck E.E. (2020). The Protective Oocyte Envelope of Threespine Stickleback Fish. http://hdl.handle.net/1773/45501.

[71] Zhang L., Ge R., Yang Y., Chen K., Li C. (2024). The zona pellucida protein piopio regulates the metamorphosis and reproduction in *Tribolium castaneum*. Arch. Insect Biochem. Physiol..

[72] Adams J.C. (2013). Evolution of the metazoan extracellular matrix. Encyclopedia of Life Sciences.

[73] Wassarman P.M., Litscher E.S. (2021). Zona pellucida genes and proteins: Essential players in mammalian oogenesis and fertility. Genes.

[74] Springate L., Frasier T.R. (2017). Gamete compatibility genes in mammals: Candidates, applications and a potential path forward. R. Soc. Open Sci..

[75] Swann C.A., Cooper S.J.B., Breed W.G. (2017). The egg coat zona pellucida 3 glycoprotein—Evolution of its putative sperm-binding region in Old World murine rodents (Rodentia: Muridae). Reprod. Fertil. Dev..

[76] Aagaard J.E., Vacquier V.D., MacCoss M.J., Swanson W.J. (2010). ZP-domain proteins in the abalone egg coat include a paralog of VERL under positive selection that binds lysin and 18-kDa sperm proteins. Mol. Biol. Evol..

[77] Baibakov B., Boggs N.A., Yauger B., Baibakov G., Dean J. (2012). Human sperm bind to the N-terminal domain of ZP2 in humanized zonae pellucidae in transgenic mice. J. Cell Biol..

[78] de la Fuente D., Maroto M., Cajas Y.N., Cañón-Beltrán K., Fernandez-Gonzalez R., Munoz-Maceda A., Sanchez-Puig J.M., Blasco R., Cots-Rodríguez P., Aviles M. (2024). Oviductin sets the species-specificity of the mammalian zona pellucida. eLife.

[79] Wassarman P.M. (2002). Sperm receptors and fertilization in mammals. Mt. Sinai J. Med. N. Y..

[80] Zhang Y., Yan Y., Li Y. (2003). Advances in studies of ZP3 binding proteins in mammalian sperm. Chin. Bull. Life Sci..

[81] Aagaard J.E., Springer S.A., Soelberg S.D., Swanson W.J. (2013). Duplicate abalone egg coat proteins bind sperm lysin similarly, but evolve oppositely, consistent with molecular mimicry at fertilization. PLoS Genet..

[82] Srivastava M., Ruiz-Trillo I., Nedelcu A.M. (2015). A comparative genomics perspective on the origin of multicellularity and early animal evolution. Evolutionary Transitions to Multicellular Life: Principles and Mechanisms.

[83] Hynes R.O. (2012). The evolution of metazoan extracellular matrix. J. Cell Biol..

